# Ovarian cancer cell-secreted exosomal miR-205 promotes metastasis by inducing angiogenesis

**DOI:** 10.7150/thno.37455

**Published:** 2019-10-18

**Authors:** Liuqing He, Wei Zhu, Quan Chen, Yishu Yuan, Yixuan Wang, Junpu Wang, Xiaoying Wu

**Affiliations:** 1 Department of Pathology, Xiangya Hospital, Central South University, Changsha, Hunan 410078, P.R. China;; 2 Department of Pathology, School of Basic Medical Science, Central South University, Changsha, Hunan 410013, P. R. China.

**Keywords:** ovarian cancer, exosomes, miR-205, angiogenesis, metastasis

## Abstract

**Background:** By providing oxygen, nutrients and metastatic conduits, tumour angiogenesis is essential for cancer metastasis. Cancer cell-secreted microRNAs can be packaged into exosomes and are implicated in different aspects of tumour angiogenesis. However, the underlying mechanisms are incompletely understood.

**Methods:** The GEPIA database and *in situ* hybridization assay were used to analyse expression of miR-205 in ovarian tissues. Immunohistochemistry was performed to examine the relationship between miR-205 and microvessel density. Expression of circulating miR-205 was evaluated by RT-PCR and GEO database analysis. Co-culture and exosome labelling experiments were performed to assess exosomal miR-205 transfer from ovarian cancer (OC) cells to endothelial cells ECs. Exosome uptake assays were employed to define the cellular pathways associated with the endocytic uptake of exosomal miR-205. The role of exosomal miR-205 in angiogenesis was further investigated *in vivo* and *in vitro*. Western blotting and rescue experiments were applied to detect regulation of the PTEN-AKT pathway by exosomal miR-205 in ECs.

**Results:** miR-205 was up-regulated in OC tissues, and high expression of miR-205 was associated with metastatic progression in OC patients. Moreover, miR-205 was highly enriched in cancer-adjacent ECs, and up-regulation of miR-205 correlated positively with high microvessel density in OC patients. Importantly, miR-205 was markedly enriched in the serum of OC patients, and a high level of miR-205 in circulating exosomes was associated with OC metastasis. In addition, OC-derived miR-205 was secreted into the extracellular space and efficiently transferred to adjacent ECs in an exosome-dependent manner, and the lipid raft-associated pathway plays an important role in regulating uptake of exosomal miR-205. Exosomal miR-205 from OC cells significantly promoted *in vitro* angiogenesis and accelerated angiogenesis and tumour growth in a mouse model. Furthermore, we found that exosomal miR-205 induces angiogenesis via the PTEN-AKT pathway.

**Conclusion:** These findings demonstrate an exosome-dependent mechanism by which miR-205 derived from cancer cells regulates tumour angiogenesis and implicate exosomal miR-205 as a potential therapeutic target for OC.

## Introduction

Angiogenesis is essential for cancer development and metastasis by providing oxygen and nutrients. Folkman demonstrated that tumours depend on the constant growth of new blood vessels, a process called angiogenesis, and that interrupting a tumour's blood supply should eliminate the cancer 1. In recent decades, many angiogenesis inhibitors have been recommended for treatment and have also been approved for many cancers 2. However, the results for many angiogenesis inhibitors are not sufficient, partly due to an incomplete understanding of tumour angiogenesis. Thus, it is important to investigate potential angiogenesis factors involved in tumour angiogenesis, which may provide a more efficient way to prevent nutrition to tumours and used for the treatment of cancer metastasis.

Exosomes, extracellular vesicles (EVs) 30 to 100 nm in diameter, are composed of a lipid bilayer and contain a variety of functional biological molecules, including proteins, microRNAs (miRNAs), and DNA 3, 4. According to recent studies, exosomes can facilitate cancer progression by mediating miRNA communication between tumour cells and surrounding cells 5, 6. miRNAs impact tumour angiogenesis by targeting angiogenic factors directly or indirectly and by regulating the biological behaviours of ECs, which are the most important effector cells in angiogenesis 7. It has been confirmed that cancer-secreted miRNAs can be transported to adjacent ECs via exosomes and exert biological effects of ECs 8. Moreover, cancer diagnosis via detection of circulating exosomal miRNAs in the serum has been proven to be a reliable method 9, 10. Therefore, strategies to block the loading, transport or uptake of exosomal miRNAs may be an effective approach for the treatment of OC. Nonetheless, how ovarian cancer cell-derived exosomal miRNAs promote OC metastasis through tumour angiogenesis regulation needs to be further investigated.

Previous studies reported that by promoting metastasis and invasion of cancer cells, miR-205 may serve as a tumour promoter in many cancers 11, but whether cancer cell-derived miR-205 can be released into the tumour microenvironment in a paracrine manner and consequently influence tumour angiogenesis has not been well investigated. Here, we demonstrate that miR-205 is associated with OC metastasis and angiogenesis. In addition, expression of circulating miR-205 is up-regulated in OC patients, and high expression of exosomal miR-205 in serum is related to OC metastasis. Importantly, cancer-derived miR-205 can be transported to ECs via exosomes, and internalization of exosomal miR-205 in human umbilical vein endothelial cells (HUVECs) involves nonclassical, lipid raft-dependent endocytosis. Further studies showed that exosomal miR-205 promotes angiogenesis *in vitro* and *in vivo* by regulating the PTEN-AKT pathway. This study identified the mechanisms by which exosomes mediate communication via miR-205 between ovarian cancer cells (OCCs) and ECs and confirmed the role of cancer-derived miR-205 in tumour angiogenesis. Thus, our work may advance our understanding of tumour angiogenesis in OC metastasis and may provide more effective treatment for OC patients with a high risk of metastasis.

## Results

### Up-regulation of miR-205 is related to metastatic progression and microvessel density in OC patients

**Figure 1:**
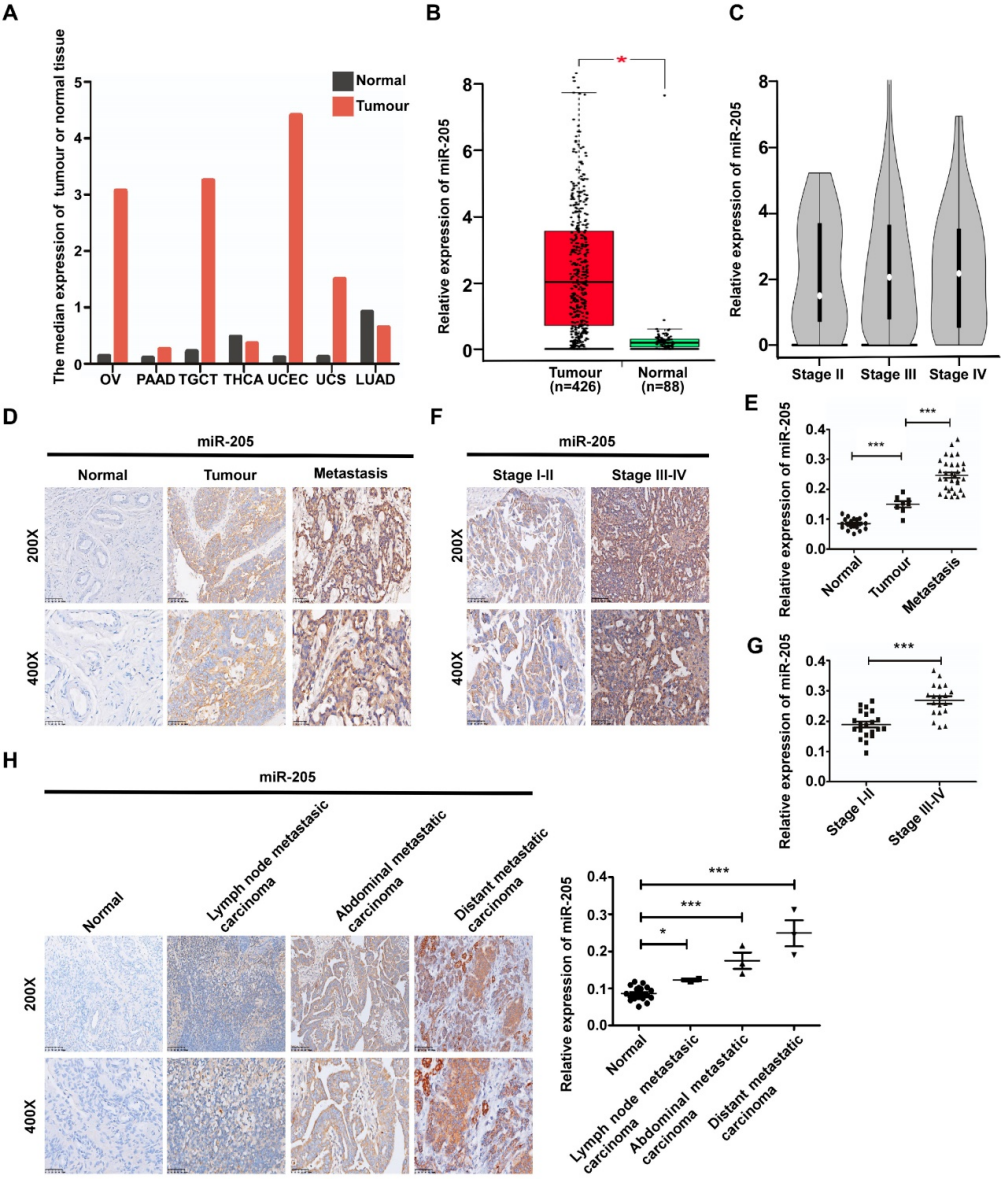
** miR-205 is up-regulated in ovarian cancer and correlates positively with metastatic progression in OC patients**. (**A**) miR-205 expression profile across TCGA datasets. Images were obtained from the GEPIA online database (http://gepia.cancer-pku.cn). OV: ovarian serous cystadenocarcinoma; PAAD: pancreatic adenocarcinoma; TGCT, testicular germ cell tumour; THCA: thyroid carcinoma; UCEC, uterine corpus endometrial carcinoma; UCS, uterine carcinosarcoma; LUAD: lung adenocarcinoma; tumour, ovarian cancer tissue; normal, normal ovarian tissue. (**B**) Expression levels of miR-205 in ovarian tissues from TCGA datasets. Tumour, ovarian cancer tissue; normal, normal ovarian tissue. (**C**) Expression levels of miR-205 in ovarian tissues from stage II, stage III and stage IV. (**D**) Representative images of miR-205 measured by ISH in tissues from normal, tumour, metastasis. Normal, normal ovarian tissue; tumour, ovarian cancer tissue; metastasis, ovarian cancer tissue with metastasis. (**E**) Statistical comparison of differences in expression of miR-205 in the three groups. (**F**) Representative images of miR-205 measured by ISH in ovarian tissues from stage I-II and stage III-IV. (**G**) Statistical comparison of differences in expression of miR-205 in the two groups. (**H**) ISH analysis and statistical comparison of differences in miR-205 expression in normal ovarian samples and metastatic carcinoma samples. The scale bar in 200× images represents 100 µm. The scale bar in 400× images represents 50 µm. All results are presented as the mean ± SEM. *P < 0.05 and ***P < 0.001, Student's *t* test.

We previously reported that miR-205 promotes metastasis and invasion in OCCs 12, but the underlying molecular mechanisms remain poorly characterized. To investigate the role of miR-205 in the metastatic progression of OC, the GEPIA database 13 was used to analyse expression of miR-205 in OC, and 68 paraffin-embedded archived ovarian tissues were collected for *in situ* hybridization (ISH). The GEPIA results showed that miR-205 is up-regulated in many types of cancers, including OC (Figure 1A, B). Compared with stage II OC tissues, the expression of miR-205 was dramatictly up-regulated in stage III-IV OC tissues (Figure 1C). Consistently, the results of ISH revealed a significantly increased level of miR-205 expression in OC tissues that was even higher in OC tissues with metastasis (Figure 1D, E). Moreover, the miR-205 level was markedly up-regulated in stage III-IV OC tissues compared with stage I-II OC tissues (Figure 1F, G). We also evaluated the expression levels of miR-205 in metastatic tissues of OC and found it to be greatly increased in metastatic carcinoma, especially in distant carcinoma, when compared with normal ovarian tissues (Figure 1H).

**Figure 2:**
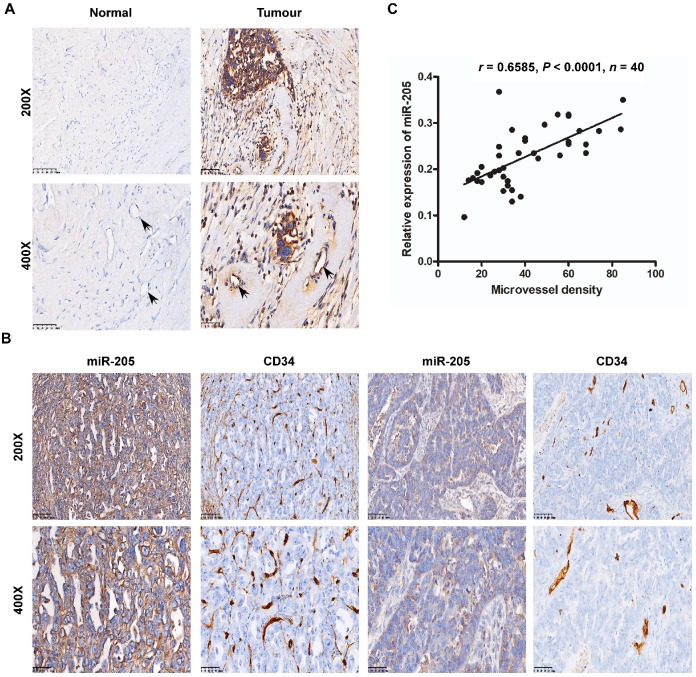
** Overexpression of miR-205 correlates positively with MVD in OC patients**. (**A**) Relative expression of miR-205 measured by ISH in ECs (black arrows). The scale bar in 200× images represents 100 µm. The scale bar in 400× images represents 50 µm. (**B**) Representative images of miR-205 IHC for CD34 with high or low expression levels of miR-205. The scale bar in 200× images represents 100 µm. The scale bar in 400× images represents 50 µm. (**C**) Spearman correlation between miR-205 expression and MVD in OC tissues. Pearson correlation coefficient (r) and *P*-value are shown, n=40.

Interestingly, our data also showed that miR-205 was expressed in cancer-adjacent ECs as well as in OCCs, whereas normal ovarian cells and their surrounding ECs were negative for miR-205 expression (Figure 2A). ECs are the most important effector cells in angiogenesis and play a significant role in tumour metastasis and development 14, and CD34, a highly glycosylated transmembrane cell surface glycoprotein, is a novel marker for ECs 15. Thus, to investigate the relationship between miR-205 and angiogenesis, ISH and immunohistochemistry (IHC) were employed to detect miR-205 and CD34 in the same two independent sets of OC specimens. According to the results, microvessel density (MVD) was significantly greater in OC patients with high miR-205 expression than in those with low expression (Figure 2B), and Spearman correlation analysis indicated a positive correlation between high expression of miR-205 and increased MVD (Figure 2C, Table S1). These results demonstrate that miR-205 is a metastasis-associated miRNA in OC and that its up-regulation correlates positively with MVD.

### Circulating exosomal miR-205 is associated with OC metastasis

**Figure 3:**
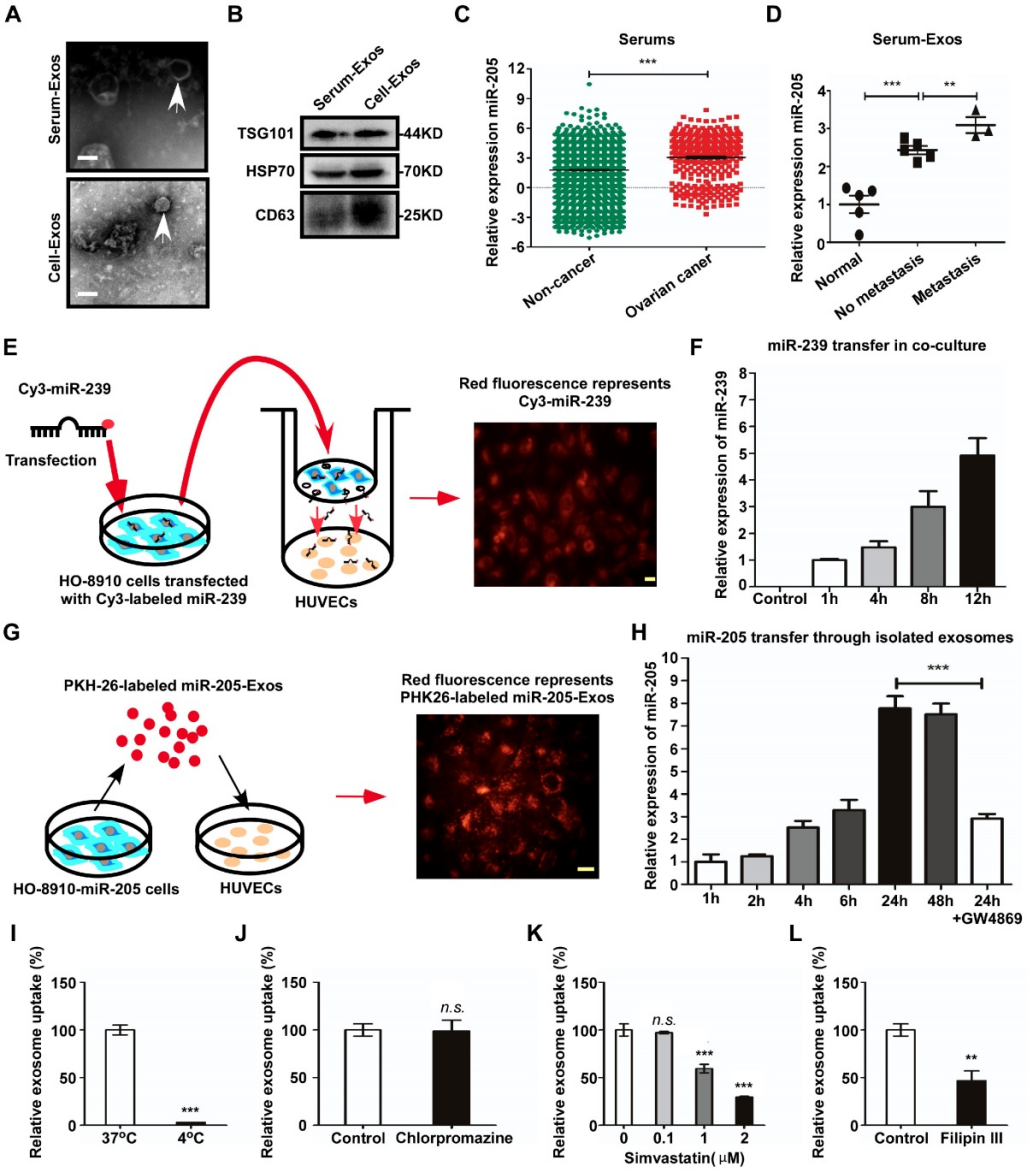
** Exosomal miR-205 can be transferred from OCCs to HUVECs and is associated with OC metastasis.** (**A**) Electron microscopy was used to analyse isolated exosomes (white arrows). Scale bar, 100 nm. (**B**) The exosome-specific marker TSG101 and extracellular vesicle-related protein markers HSP70 and CD63 were measured by western blotting analysis. (**C**) Relative expression levels of miR-205 in serum were obtained from serum microRNA profiles (GSE106817 database), which consist of 333 ovarian cancers and 2759 non-cancer controls. (**D**) RT-PCR was used to detect miR-205 levels in exosomes isolated from the serum of OC patients. (**E**) HO-8910 cells transfected with a Cy3-labeled miR-239 mimic were co-cultured with HUVECs in a transwell device. The scale bar represents 20 µm. (**F**) HO-8910 cells were transfected with cel-miR-239 or left untransfected and RNA was isolated from HUVECs at different times after the start of the co-culture. Levels of cel-miR-239 in HUVECs were measured by RT-PCR. (**G**) Exosomes from HO-8910-miR-205 cells were labelled with PKH26 and then added to HUVEC culture medium. The scale bar represents 20 µm. (**H**) The effects of GW4869 on exosome-dependent miR-205 delivery from OCCs to recipient HUVECs were observed by RT-PCR. (**I**) HUVECs were used to investigate uptake of exosomal miR-205 at 4 °C. The relative uptake of exosomal miR-205 was analysed in the presence of various uptake inhibitors, including chlorpromazine (**J**), simvastatin (**K**) and filipin III (**L**). All data are shown as the mean ± SEM from at least three independent experiments. **P < 0.01 and ***P < 0.001, Student's *t* test. *n.s*, not significant.

To evaluate the relationship between exosomal miR-205 and OC, exosomes were isolated from the serum of OC patients (Serum-Exos) or from the conditioned medium of OCCs (Cell-Exos). Transmission electron microscopy (TEM) showed an exosome teacup-like double-sided structure of 30-100 nm (Figure 3A). In addition, the specific endosomal markers HSP70, TSG101 and CD63 16, 17 were examined (Figure 3B). Recently, Taylor et al. 9 proposed a method to diagnose OC by detecting circulating exosomal miRNAs in serum and found exosomes derived from OC patients to be specifically enriched in miR-205. To confirm whether circulating miR-205 is associated with OC progression, expression of circulating miR-205 in the serum of OC patients was examined using the GEO database (GSE106817 18). The results showed significantly higher levels of miR-205 in OC patients than in non-cancer patients (Figure 3C). To validate these findings, RT-PCR was applied to detect the expression levels of miR-205 in circulating exosomes. Consistently, we found that serum exosomal miR-205 was specifically increased in OC patients and that the expression levels of miR-205 were dramatically up-regulated in OC patients with metastasis compared with those without metastasis (Figure 3D), which was consistent with our previous findings for OC paraffin-embedded tissues. Taken together, these results demonstrate that circulating miR-205 is significantly up-regulated in OC patients and that high expression of circulating exosomal miR-205 is associated with OC metastasis.

### OCC-secreted miR-205 is transferred to ECs via exosomes in which endocytic uptake is mediated by a lipid raft-associated pathway

Many studies have shown that miRNAs play an important role in angiogenesis and can be transferred from cell to cell via exosomes 19. Given that high expression of circulating exosomal miR-205 is related to OC metastasis, we investigated whether exosomes mediate the transfer of miR-205 from OCCs to ECs. First, we determined whether OCC-derived miRNAs can be transported to ECs in a paracrine manner using a co-culture system to prevent direct cell contact and the transfer of larger vesicles 20. We transfected HO-8910 cells with a fluorescent Cy3-labelled miR-239 that is naturally present only in *Caenorhabditis elegans* (cel-miR-239) and assessed Cy3 levels by fluorescence microscopy after 3 days of co-culture (Figure 3E). The appearance of red fluorescence (Cy3 expression) in the HUVECs indicated that Cy3-miR-239 was transferred from the HO-8910 cells in the upper Transwell chamber to the HUVECs in the lower chamber (Figure 3E). In addition, RT-PCR results showed that expression of miR-239 in the HUVECs was markedly increased in a time-dependent manner (Figure 3F). These results show that miRNAs can be secreted into the extracellular space by OCCs and transported into HUVECs.

Next, we quantified relative expression of miR-205 in four OC cell lines, including HO-8910pm, HO-8910, SKOV-3ip and SKOV-3, and their exosomes, and the results showed that exosomal miR-205 expression was consistent with cellular miR-205 expression (Figure S1A). Previous studies have reported that VEGF advances OC progression and up-regulates the expression levels of miR-205 in OC cell lines 12, 21. To further investigate whether up-regulation of miR-205 in cells can affect miR-205 expression levels in exosomes, HO-8910 and SKOV-3 cells were treated with VEGF_165_. After 24 h of treatment with VEGF_165_ (50 ng/mL), levels of miR-205 in both exosomes and cells were significantly increased (Figure S1B).

We then investigated whether OCC-secreted exosomal miR-205 can be taken up by HUVECs. We used the red fluorescent dye PKH26 to label high-miR-205-containing exosomes (miR-205-Exos), which were isolated from HO-8910 cells stably overexpressing miR-205 (HO-8910-miR-205), and analysed miR-205 expression at different times after the addition of the exosomes to the culture medium (Figure 3G). The results indicated that these high-miR-205-containing exosomes were transferred from OCCs to HUVECs (Figure 3G) and increased the expression levels of miR-205 in a time-dependent manner (Figure 3H). Consistently, GW4869, an exosome generation inhibitor, attenuated exosome production and the transfer of miR-205 to HUVECs, indicating that this transfer mainly depended on exosomes (Figure 3H).

Additionally, exosome uptake by HUVECs was completely abolished at 4 °C (Figure 3I), showing that exosome uptake is an active process. We next investigated the cellular pathways associated with the uptake of exosomal miR-205 and found that chlorpromazine, a specific inhibitor of clathrin-mediated endocytosis, had no effect on exosome uptake relative to the control (Figure 3J). To determine whether exosomal miR-205 uptake requires intact lipid membrane rafts, we performed experiments with simvastatin, which acts by inhibiting the rate-limiting enzyme of cholesterol biosynthesis, 3-hydroxyl-3-methylglutaryl coenzyme A (HMG-CoA) reductase, and reduces intracellular cholesterol levels in HUVECs 22. The results showed that simvastatin dose-dependently suppressed OCC-derived exosomal miR-205 internalization in HUVECs (Figure 3K). Lipid raft-associated pathways, also defined as non-classical endocytosis pathways, can be either dependent or independent of caveolin-1 (CAV1) 23. Notably, filipin III, an inhibitor of lipid raft-dependent and caveolar endocytosis, was shown to reduce exosomal miR-205 uptake (Figure 3L). In summary, these experiments demonstrate that OCCs secrete miR-205, which is then efficiently taken up by ECs via exosomes in a non-classical endocytosis pathway.

### Exosomal miR-205 induces angiogenesis *in vitro* and *in vivo*

**Figure 4:**
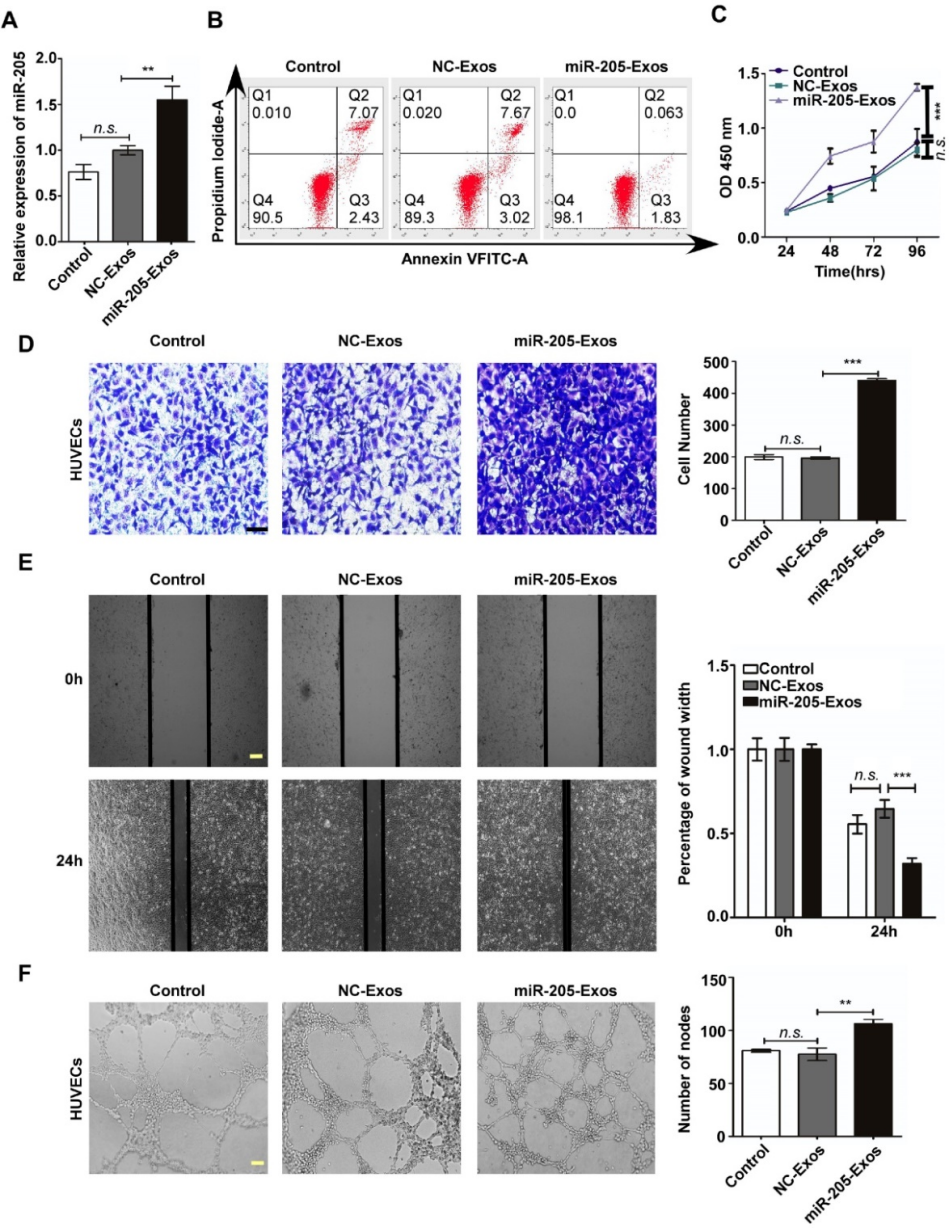
** Exosomal miR-205 promotes *in vitro* angiogenesis.** (**A**) After treatment with miR-205-Exos, NC-Exos and control (as blank control), the miR-205 levels in HUVECs were measured by RT-PCR. (**B**) Apoptosis analysis was performed after treatment with different exosomes. (**C**) The viability of HUVECs treated with various exosomes was assessed by the CCK-8 assay. (**D**) Transwell assays ere performed to measure the migration of HUVECs treated with various exosomes. The scale bar represents 50 µm. (**E**) The wound closure assay was performed to detect cell migration. The scale bar represents 100 µm. (**F**) The tube formation assay was conducted using HUVECs treated with exosomes and Matrigel. The scale bar represents 100 µm. The mean ± SEM is provided (n=3). *n.s*, not significant. **P < 0.01 and ***P < 0.001, Student's *t* test.

**Figure 5:**
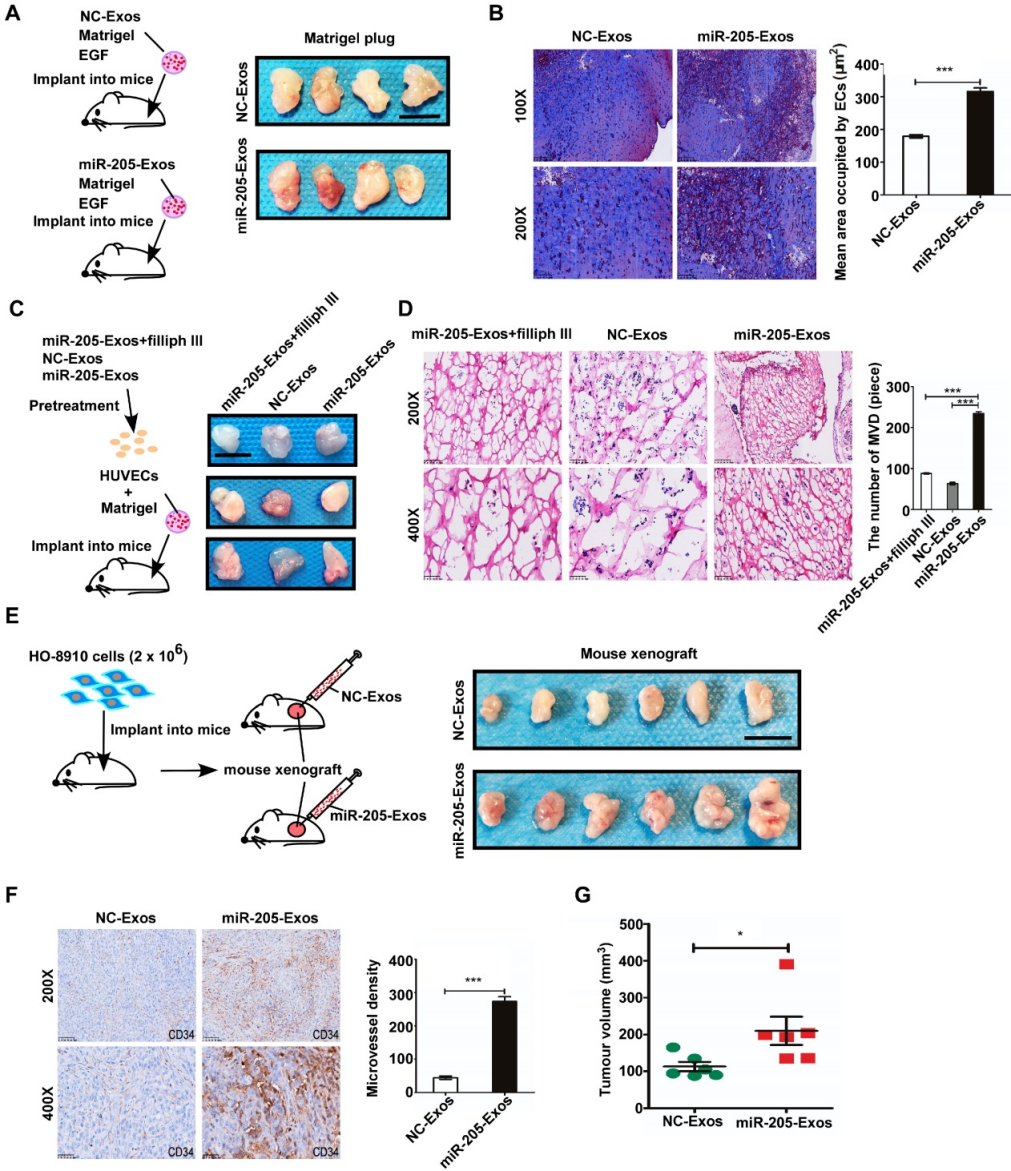
** Exosomal miR-205 enhances *in vivo* angiogenesis.** (**A**) Matrigel mixed with miR-205-Exos or NC-Exos and EGF was subcutaneously implanted into mice (n = 4 each) and then harvested 10 days after inoculation. Scale bar, 10 mm. (**B**) Sections of the Matrigel plugs were stained with Masson's trichrome, which stains Matrigel blue and endothelial cells red. All sections are oriented with the side underlying the skin at the bottom of the figure. (**C**) Mixture containing Matrigel and HUVECs was subcutaneously implanted into mice (n = 3 each) and then harvested 7 days after inoculation. Scale bar, 10 mm. (**D**) Frozen slices of the Matrigel plugs were stained with eosin-haematoxylin. (**E**) After 7-9 days, subcutaneous mouse xenografts were intratumourally injected with miR-205-Exos or NC-Exos (n=6 each). Scale bar, 10 mm. (**F**) After injection with exosomes, mouse xenografts were subjected to IHC staining for CD34 and quantitatively assessed for angiogenesis. (**G**) The difference in tumour volumes between the miR-205-Exos treatment group and NC-Exos treatment group was statistically significant. The scale bar in 100× images represents 200 µm. The scale bar in 200× images represents 100 µm. The scale bar in 400× images represents 50 µm. All data are the mean of biological replicates from a representative experiment, and the results are presented as the mean ± SEM. *P < 0.05 and ***P < 0.001, according to two-tailed Student's t test.

To reduce the effect of other factors of exosomes, a model system was used to alter exosomal miR-205 expression. First, we used HO-8910 cells infected with miR-205-overexpressing lentivirus or NC-control vector lentivirus to establish stably expressing miR-205 cells. Next, we isolated low-miR-205-containing (NC-Exos) or high-miR-205-containing (miR-205-Exos) exosomes and confirmed expression of miR-205 by RT-PCR (Figure 4A). Furthermore, exosomal miR-205 markedly promoted proliferation, invasion and migration in OCCs in a manner similar to that of endogenous miR-205 (Figure S2). We then carried out a series of experiments to further determine whether exosomal miR-205 induces angiogenesis. Compared with the control or NC-Exos, miR-205-Exos notably increased miR-205 expression in HUVECs and promoted *in vitro* angiogenesis-related processes, including HUVEC proliferation (Figure 4B, C), migration (Figure 4D, E) and tube formation (Figure 4F). Because exosomal miR-205 enhances angiogenesis *in vitro*, a Matrigel plug assay was first performed to detect whether it can regulate angiogenesis *in vivo* (Figure 5A). Compared with NC-Exos, plugs containing miR-205-Exos exhibited more efficient recruitment of ECs (Figure 5B), suggesting that exosomal miR-205 efficiently enhances angiogenesis *in vivo*. To further study whether an uptake inhibitor affects MVD, HUVECs pretreated with filipin III or exosomes were fixed with Matrigel and then separately implanted into mice (Figure 5C). As shown in Figure 5D, miR-205-Exos markedly increased MVD relative to the control, whereas these effects were reversed by treatment with filipin III. A mouse xenograft model was then established to assess *in vivo* angiogenesis (Figure 5E). Consistently, mice xenografts with miR-205-Exos treatment led to a higher MVD (Figure 5F) and larger tumour size (Figure 5G) than did NC-Exos treatment. These results suggest that exosomal miR-205 dramatically enhances angiogenesis both *in vitro* and *in vivo*. Indeed, these findings indicate that OCC-derived miR-205 induces angiogenesis via exosomes.

### Exosomal miR-205 promotes angiogenesis by regulating the PTEN-AKT pathway

**Figure 6:**
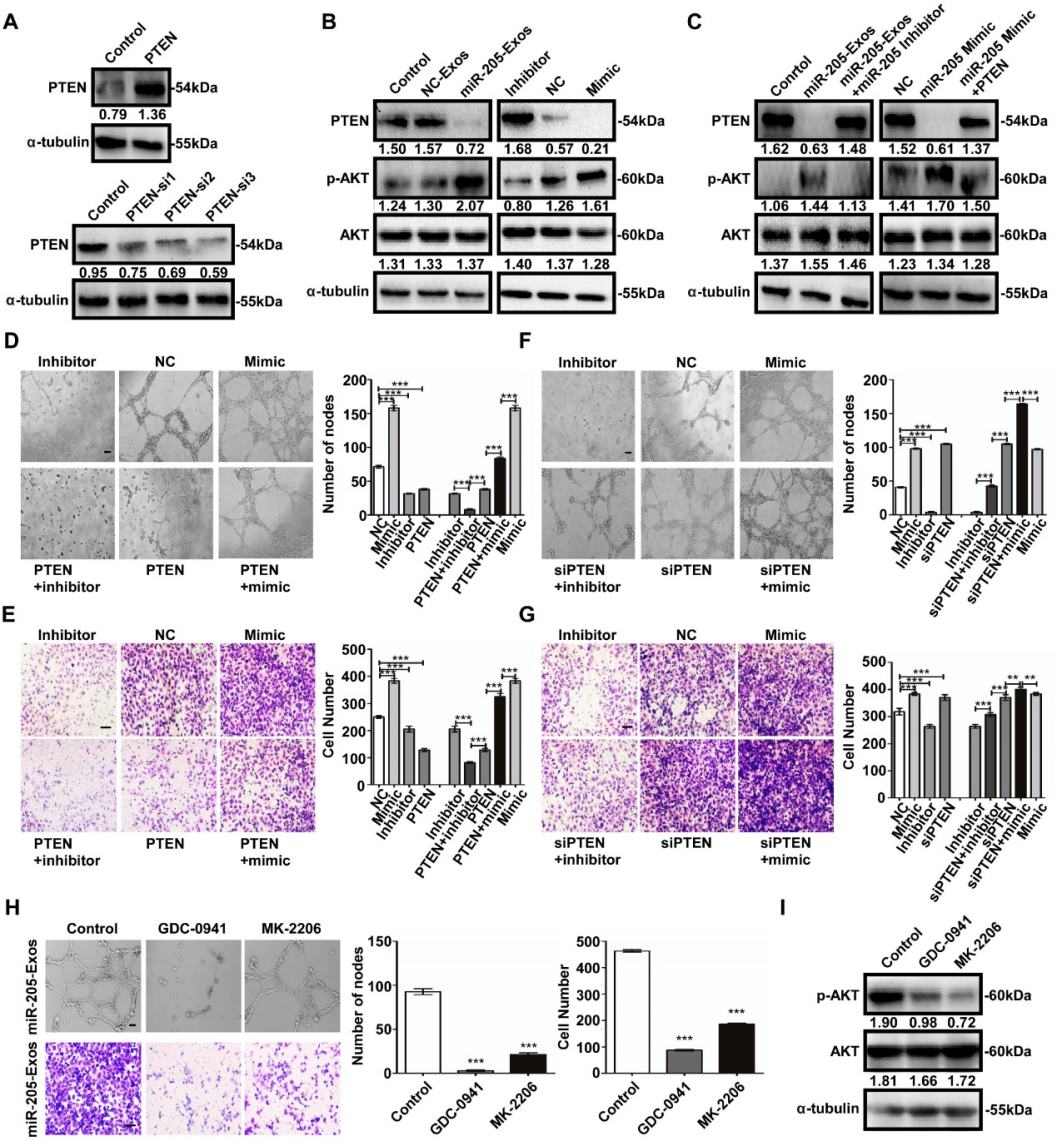
** Exosomal miR-205 regulates the PTEN-AKT signalling pathway in HUVECs.** (**A**) Interference efficiency was measured by western blotting. (**B**) Western blotting analysis of PTEN, p-AKT, AKT, and α-tubulin in HUVECs incubated with NC-Exos, miR-205-Exos, miR-205 inhibitor, NC and mimic groups. (**C**) Western blotting was used to detect PTEN, p-AKT and AKT expression in HUVECs after treatment with miR-205-Exos, miR-205-Exos+miR-205 inhibitor, NC, miR-205 mimic and miR-205 mimic+PTEN. (**D, E**) Effects of miR-205 inhibitor, NC, mimic, PTEN+inhibitor, PTEN, PTEN+mimic on HUVEC tube formation ability and migration were assessed by tube formation and Transwell migration assays. (**F, G**) Effects of miR-205 inhibitor, NC, mimic, siPTEN+inhibitor, siPTEN, siPTEN+mimic on HUVEC tube formation ability and migration were examined by tube formation and transwell migration assays. The scale bar in (D, F) represents 100 µm. The scale bar in (E, G) represents 50 µm. (**H**) Effects of GDC-0941 and MK-2206 on tube formation and migration abilities induced by exosomal miR-205 in HUVECs. The scale bar in the upper panel represents 100 µm. The scale bar in the bottom panel represents 50 µm. (**I**) Western blot analysis shows the effects of GDC-0941 and MK-2206. All experiments were repeated three times, and the results are presented as the mean ± SEM. Statistical significance was determined by a two-tailed, unpaired Student's *t* test. **P < 0.01 and ***P < 0.001.

To determine the mechanism by which exosomal miR-205 modulates angiogenesis, a large number of potential target genes for miR-205 were predicted using TargetScan and miRBase, and PTEN was chosen for further study due to its suppression of angiogenesis. We examined the role of miR-205-mediated inhibition of PTEN. PTEN mRNAs contain a possible binding site for miR-205 (Figure S3A), and suppression of miR-205 by PTEN was confirmed by luciferase reporter gene assays in previously work 12. Notably, ectopic miR-205 expression in HUVECs resulted in marked decreases in PTEN mRNA levels (Figure S3B), indicating that miR-205 indeed represses PTEN expression in HUVECs by directly targeting the 3' UTR of the PTEN mRNA. In addition, miR-205 expression at different levels was associated with changes in angiogenic potential (Figure S4). To confirm whether exosomal miR-205 can induce angiogenesis through the PTEN-AKT pathway, HUVECs were treated with miR-205-Exos, NC-Exos, miR-205 mimic, inhibitor, or NC, PTEN or PTEN small-interfering RNA (siRNA). After transfection of HUVECs with the PTEN expression plasmid or PTEN siRNA, protein expression of PTEN was up- or down-regulated, respectively (Figure 6A). Notably, treatment with miR-205-Exos or miR-205 mimic resulted in significantly decreased expression of PTEN and increased phosphorylated-AKT (p-AKT) in HUVECs (Figure 6B), and these effects were reversed by miR-205 inhibitor or PTEN expression (Figure 6C). In addition, miR-205-Exos reduced the protein levels of PTEN and E-cadherin in OCCs (Figure S5), and the enhanced tube formation and cell migration due to miR-205 overexpression was blocked by PTEN expression (Figure 6D, E).

Similarly, the tube formation and cell migration suppressed by miR-205 inhibition in HUVECs was rescued by treatment with PTEN siRNA (Figure 6F, G). We next evaluated the effect of specific blockade of PI3K/AKT pathways on the migration-related and angiogenic functions of exosomal miR-205. Treatment with the PI3K inhibitor GDC-0941 or AKT inhibitor MK-2206 drastically reduced the migration and tube formation abilities induced by exosomal miR-205 in HUVECs (Figure 6H), and as shown in Figure 6I, the effectiveness of GDC-0941 or MK-2206 treatment was confirmed. Collectively, these results suggest that exosomal miR-205 derived from OCCs modulates angiogenesis by regulating PTEN-AKT signalling.

## Discussion

Previous studies have indicated that miR-205 promotes invasion and metastasis in OCCs and is associated with poor prognosis in OC. However, it remains unknown whether cancer-derived miR-205 can be delivered to cancer-adjacent ECs and thus impact metastatic progression of OC. In this study, we showed that miR-205 is associated with OC metastasis and can be transferred from OCCs to ECs via exosomes in which endocytic uptake is mediated by the lipid raft-associated pathway. In addition, miR-205 was significantly enriched in the serum of OC patients, and high expression of circulating miR-205 was associated with OC progression. Further analyses showed that exosomal miR-205 induced angiogenesis *in vivo* and *in vitro* through the PTEN-AKT pathway. Taken together, these data elucidate the role of OCC-derived exosomal miR-205 in OC metastasis and the underlying mechanisms responsible for miR-205-associated metastasis.

Invasion and metastasis are significant biological behaviours of tumour cells and closely related to poor prognosis 24, seriously threatening the health and life of patients with metastasis 25, 26. Therefore, in-depth analysis of the mechanisms of metastasis is important to improve the survival rate and prognosis of OC patients. miR-205 has been reported to function as a cancer-promoting miRNA in many cancers 27, and GEPIA database results showed that miR-205 is up-regulated in ovarian cancer. Consistently, our ISH results demonstrated that miR-205 is significantly increased in OC specimens and positively associated with OC metastasis.

The growth, invasion and metastasis of tumours depend on angiogenesis, which provides sufficient oxygen and nutrition for tumour cells. Thus, drugs that aim to eliminate the supply of nutrients to tumours, known as angiogenesis inhibitors, have been hailed as opening a new era in cancer therapy 2. Indeed, targeting the blood vessels that feed tumours has been widely accepted as a clinical therapeutic strategy in recent years. However, a large number of reports suggest that such drugs have yet to show significant benefits in patients 28, partly due to an incomplete understanding of tumour angiogenesis. Here, we explore the role of miR-205 in OC metastasis as a potential angiogenesis factor. According to recent studies, high MVD is more likely to occur in patients with metastasis 29. Our ISH and IHC assays revealed that miR-205 expression in OCCs and cancer-adjacent ECs was positively associated and that up-regulation of miR-205 correlated positively with high MVD in OC patients. Overall, these results confirm that miR-205 is involved in angiogenesis and may provide another explanation for miR-205 up-regulation-related OC metastasis.

Recent studies have shown that high levels of miRNAs in circulating exosomes are related to cancer progression, and many studies have asserted that cancer diagnosis via detection of circulating miRNAs in serum can be used as a reliable blood-based biomarker for cancer diagnosis 9,10. The results of our GEO database and RT-PCR analyses demonstrated that miR-205 expression is significantly increased in the serum of OC patients and those high expression levels of circulating exosomal miR-205 correlate with OC metastasis.

Many miRNAs have been reported to play significant roles in pathological or physiological processes, including angiogenesis. Moreover, numerous studies have revealed that exosomes may serve as a novel transporter that can transfer miRNAs from cell to cell 30, 31. Combining these studies, our previous results led us to hypothesize that miR-205 may be secreted by OCCs and then transported to adjacent ECs via exosomes, and several assays were carried out to test our hypothesis. First, HO-8910 cells were transfected with Cy3-miR-239 and then co-cultured with HUVECs, showing a several-fold increase of red fluorescently labelled miR-239 in HUVECs. These data demonstrate that miRNA can be transferred from OCCs to HUVECs during co-culture. Due to their endogenous actions, miRNAs in cells can be packaged into exosomes and secreted to the extracellular space. Indeed, expression of miR-205 was higher in exosomes collected from OCCs with high levels of miR-205 but lower in exosomes derived from OCCs with low levels of miR-205, indicating that miR-205 in cells can be packaged into exosomes. To further confirm whether up-regulation of miR-205 in cells can affect the expression levels of miR-205 in exosomes, VEGF was used to induce increase levels of miR-205 in OCCs. Our data showed that VEGF_165_ significantly increased the expression and secretion of miR-205, which was enriched in exosomes; therefore, up-regulation of miR-205 in cells definitely increases the expression levels of miR-205 in exosomes. According to a recent study, cancer cell-derived exosomal miRNAs can be transported to other cell types 32, and we found that fluorescently labelled miR-205-containing exosomes were taken up by HUVECs and increased the levels of miR-205 in a time-dependent manner. Consistently, this was accompanied by increased expression of miR-239 in HUVECs, and these effects were suppressed by GW4869, which inhibits exosome generation. Furthermore, we investigated the specific signalling mechanisms involved in the uptake process of exosomal miR-205 and found a significant role for lipid raft-associated endocytosis in regulating the uptake of exosomal miR-205 by HUVECs. Here, we confirm that OCC-secreted miR-205 via exosomes can be efficiently taken up by ECs in the lipid raft-associated endocytosis pathway.

Cancer cell-secreted miRNA-containing exosomes, which can be taken up by neighbouring or distant recipient cells to promote tumour development and transfer, play an important role in tumour angiogenesis. For example, metastasis-associated exosomal miR-23a is transferred from nasopharyngeal carcinoma (NPC) cells for HUVEC-mediated angiogenesis in NPC 33. Exosome-mediated miR-25-3p derived from colorectal cancer (CRC) cells can be transported to ECs and induce angiogenesis, consequently promoting pre-metastatic niche formation 34. Thus, to explore whether exosomal miR-205 regulates angiogenesis, a series of experiments were carried out. After treatment with exosomal miR-205, cell proliferation, migration and tube formation abilities of ECs were dramatically increased, suggesting that OCC-derived miR-205 promotes *in vitro* angiogenesis via exosomes. *In vivo* angiogenesis experiments also confirmed that exosomal miR-205 accelerates the formation of blood vessels in the model of Matrigel thrombus and consistently promotes tumour angiogenesis in a subcutaneous tumour model. In addition, filipin III had a negative effect on MVD changes induced by exosomal miR-205. These findings demonstrate the important role of OCC-secreted miR-205 in the metastasis of OC and the mechanism by which miR-205 promotes tumour angiogenesis in a unique way mediated by exosomes.

To explore the mechanism, we screened miR-205 target genes, with PTEN being implicated owing to its function in suppressing angiogenesis. PTEN is a key mediator of the PI3K/AKT pathway, which plays an essential role in the formation of normal blood vessels during development 35. Frequent activation of AKT signalling can result in uncontrolled proliferation and neoplastic angiogenesis, and PTEN is a phosphatase necessary for the specific and effective termination of oncogenic AKT signalling induced by oncogenes 36, 37. According to our results, ectopic expression of miR-205 dramatically decreased PTEN both at the mRNA and protein levels and increased that of p-AKT in HUVECs. In contrast, knockdown of miR-205 blocked these effects. Analogously, restoration of PTEN expression levels rescued the suppression of angiogenesis induced by miR-205 inhibitor, revealing that miR-205 induces angiogenesis by silencing PTEN and subsequently activates its downstream AKT pathway. Importantly, PI3K or AKT inhibitor significantly retarded the cell migration and tube formation induced by exosomal miR-205. Taken together, these findings illustrate the angiogenic function of exosomal miR-205 and its contribution to the direct inhibition of PTEN in ECs as well as the activation of the AKT signalling pathway, suggesting a novel mechanism for exosomal miR-205 associated with metastasis in OC.

## Materials and Methods

### Human tissue samples

Written informed consent was obtained from all participants before specimen collection. According to the current ethical guidelines, this study was approved by the ethics review committee of Xiangya Hospital of Central South University (CSU) and the ethics review committee of the tumour hospital affiliated with Xiangya Medical College of CSU. Sixty-eight paraffin-embedded tissue samples, including 40 OC tissue specimens, 20 normal ovarian tissue specimens and 8 metastatic tissues, were collected from patients with OC admitted to Xiangya Hospital of CSU between 2014 and 2018, and the relevant prognostic information was collected for all the patients (Table S2). All specimen diagnoses were confirmed by a qualified pathologist after surgery. OC serum samples were obtained from 5 healthy volunteers and 8 OC patients who underwent tumour resection on the day in Xiangya third Hospital and the Tumour Hospital of CSU (Table S3). All the blood samples were centrifuged at 3,000 × g for 10 min at 4 °C to for serum extraction. None of the patients received any anticancer treatment prior to the operation.

### Cell lines and cell culture

HUVECs, human OC cell lines HO-8910 and SKOV-3 were purchased from China Center for Type Culture Collection (CCTCC) and CRC/PUMC (Cell Resource Center, IBMS, CAMS/ PUMC). The human OC cell lines HO-8910PM and SKOV-3ip were kindly donated by Professor Xin Lu (Obstetrics and Gynecology Hospital, Fudan University, Shanghai, China). HO-8910 cells stably overexpressing miR-205 (HO-8910-miR-205 cells) and negative control HO-8910 cells (HO-8910-NC cells) were established by the Xiaoying Wu research group of the pathology laboratory of CSU, and the transfection efficiency was verified in a previous study 12. HO-8910PM and SKOV-3ip cells were cultured in Dulbecco's modified Eagle medium (DMEM) supplemented with 10% foetal bovine serum (FBS) (GIBICO, Gaithersburg, MD, USA). HUVECs, HO-8910 and SKOV-3 cells were cultured in 1640 supplemented with 10% FBS.

### ISH

ISH was performed on OC tissue samples using an LNA™ microRNA ISH miR-205 optimization kit (Exiqon; Woburn, MA, USA). According to the manufacturer's directions, paraffin-embedded tissue samples were cut into 5-mm sections and placed on slides in fresh xylene for 15 min. Next, the slides were hydrated through ethanol solutions and 2× saline sodium citrate (SSC) for 1 min in each solution. The slides were incubated with Proteinase K solution at 37 °C for 20 min and then washed in phosphate-buffered saline (PBS). After dehydration, each slide was treated with 20 µL of Hyb/probe solution and incubated at 4 °C overnight, after which 100 µL of Hyb/probe solution was added to each, and a coverslip was immediately mounted using HybridSlip. The next day, the slides were washed in 2× SSC at 37 °C for 30 min. After incubation in the blocking buffer, the slides were incubated with an anti-digoxigenin antibody in a humid chamber at 37 °C for 60 min, treated with NBT: BCIP and stained with nuclear fast red. The stained slides were scanned and photographed using digital pathological scanning equipment. Image-Pro plus (IPP, version 5.0, Media Cybernetics, Silver Spring, MD) was employed to analyse the images according to a previous study 38. The positivity rate was calculated as the integral optical density (IOD)/area sum. The final score obtained from the IPP analysis was used to identify the relative level of miR-205 expression.

### IHC

IHC was described previously 21 and was performed using an anti-CD34 antibody (1:200; ab81289; Abcam, Cambridge, MA, USA). MVD probed by the endothelial cell marker CD34 was evaluated according to a previously described method 39. In brief, sections of tissue were screened at low magnification (40 ×) to identify areas with high MVD. Next, the stained microvessels were counted at high magnification (200 ×) in five fields. Any CD34-stained individual cell or cell cluster was counted as one microvessel. The average number of microvessels per field is presented as the MVD.

### Exosome isolation, characterization and treatment

Exosomes were purified from OCC-derived conditioned media or sera from OC patients by ultracentrifugation or exosome isolation kit methods. After 48 h of culture, conditioned medium containing 2% exosome-depleted FBS was collected. The collected biofluid was centrifuged (Beckman Coulter, Brea, CA, USA) at 4,000 × g for 10 min at 4 °C to remove cell debris and then at 17,000 × g at 4 °C for 60 min to remove remaining macropolymers. The supernatant was passed through a 0.22-µm filter (Millipore, MA, USA) and further ultracentrifuged at 200,000 × g at 4 °C for 60 min to collect vesicles smaller than 100 nm and protein aggregates. The pellets were resuspended in PBS and ultracentrifuged at 200,000 × g for 60 min at 4 °C to eliminate contaminant proteins and then dissolved in ice-cold PBS for further analysis 40. Regarding the exosome isolation kit method, the collected biofluid was centrifuged at 4,000 × g for 10 min at 4 °C to remove cell debris and then passed through a 0.22‑µm filter. After centrifugation at 3,000 × g for 30 min at 4 °C in the dialysis tube (Merck KGaA, Darmstadt, Germany), the supernatant was incubated with ExoQuick‑TC™ exosome precipitation solution (System Biosciences, Palo Alto, CA, USA) for 30 min (sera) or 6 h-overnight (media) at 4 °C. Subsequently, the mixture was centrifuged at 15,279 × g for 1 h at 4 °C to harvest the yellow exosome pellets. The characterization of the exosomes was verified by detecting expression of the EV-associated protein markers Hsp70 (1:500; ab2787; Abcam) and CD63 (1:500; ab134045; Abcam) and the exosome-specific marker TSG101 (Abcam) by western blotting analysis. To monitor exosome trafficking, a PKH26 fluorescent cell linker kit (Sigma-Aldrich, Merck KGaA) was used to label exosomes with fluorescent PKH26. Next, the PKH26-labelled exosomes were washed in PBS and centrifuged at 100,000 × g for 20 min at 4 °C to collect the exosomes, which were resuspended in PBS for further assays. For TEM, exosomes were fixed with 1% glutaraldehyde in PBS and then spotted onto 300-mesh carbon/formvar-coated grids. The grids were stained for contrast using uranyl acetate in water for 10 min and imaged by TEM (FEI, Hillsboro, OR, USA). The amount of exosomes was measured using the bicinchoninic acid (BCA) protein assay kit (Novagen, Merck Group, Madison, USA). For cell treatment, 2 × 10^5^ recipient cells were incubated with 2 µg exosomes for 48 h.

### Co-culture, exosome secretion inhibition and uptake assay

A co-culture assay was performed as described previously 20. Before starting the assay, HO-8910 cells (200,000) were seeded in 6-well Transwell plates (0.4 µm pore-size filter, Corning, Sigma-Aldrich) and cultured in complete DMEM 24 h. HUVECs (100,000) were seeded in 6-well plates and cultured in complete DMEM 24 h before the co-culture assay. After transfection with the Cy3-miR-239 mimic (RiboBio, Guangzhou, China), the HO-8910 cells were co-cultured with HUVECs for the indicated times. The appearance of Cy3 red fluorescence in HUVECs was detected after washing with PBS. For exosome secretion inhibition, HUVECs were incubated with exosomes isolated from HO-8910-miR-205 cells for the indicated times. HO-8910-miR-205 cells were also pretreated with GW4869 (10 µM, Cayman Chemical, Ann Arbor, Michigan, USA), a type of exosome generation inhibitor that can inhibit exosome secretion 41, for 24 h before exosome isolation. To investigate the endocytic uptake mechanism of exosomal miR-205, uptake was assessed in the presence of various uptake inhibitors purchased from APExBIO (Shanghai, China). HUVECs were pretreated with chlorpromazine (10 μM), simvastatin and filipin III (1.5 μM) at 37 °C for 30 min, after which miR-205-Exos (20 μg/mL) was added. Another batch of HUVECs was used to investigate the uptake of exosomal miR-205 at 4 °C. Cellular uptake of exosomal miR-205 was interpreted based on changes in miR-205 expression.

### RNA interference and vector transfection

Cy3-labelled miR-239 mimic (50 nM), miR-205 mimic (50 nM), inhibitor (100 nM) and negative control (50 nM), as well as the siRNA for PTEN (50 nM) and its negative control (50 nM) were transfected using riboFECT CP tranfection kit (RiboBio) according to the manufacturer's protocol. HUVECs (2 × 10^5^) were transfected with Flag-PTEN (2 μg, GeneCopoepia Biosciences, Shanghai, China) using Lipofectamine 2000 (1 μg/mL, Invitrogen, Carlsbad, CA). After 24 h of treatment, the transfection efficiencies were verified by fluorescence microscopy and RT-PCR analysis. The sequences used in this study are listed in Supplementary Table 4.

**RNA isolation and RT-PCR**RNA isolation and RT-PCR were performed as previously described 42. For serum exosomal RNA, all blood samples were centrifuged at 3,000 × g for 10 min at 4 °C to remove cells and debris. Next, the supernatants were used to isolate serum exosomal RNA with SeraMir Exosome RNA Amplification Kit (System Biosciences) according to the manufacturer's instructions. Total RNA was extracted from cells using the TRIzol Plus RNA purification kit (Thermo Fisher Scientifc, Inc., Waltham, MA, USA ). Reverse transcription was performed using All‑in‑One^TM^ miRNA First‑Strand cDNA Synthesis Kit (GeneCopoeia Biosciences) or All‑in‑One^TM^ First‑Strand cDNA Synthesis Kit (GeneCopoeia Biosciences) according to the manufacturer's protocol. The expression level of miR-205 or PTEN mRNA was measured by RT-PCR. U6 or GAPDH was used as a normalization control for qualification of cellular miRNA, and cel-miR-39 served as an invariant control in exosomal miRNA measurements. Real-time PCR was performed with an ABI‑7500 machine (Applied Biosystems; Thermo Fisher Scientific, Inc.). The sequences used in this study are listed in Supplementary Table 4.

### Western blotting

Cells and exosomes were lysed in lysis buffer containing protease inhibitor and quantified using a BCA protein assay kit (Novagen). Subsequently, a 30-µg sample of denatured protein (for each sample) was subjected to SDS-PAGE and transferred onto PVDF membranes (Millipore). The membranes were blocked with 5% skimmed milk for 2 h at room temperature and incubated overnight at 4 °C with the follows primary antibodies: anti-phosphoAKT (ser473) (1:500; no. 4060; Cell Signaling Technology, Danvers, MA, USA), anti-AKT (1:500; no. 4691; Cell Signaling Technology) and anti-PTEN (1:500; no. 9188; Cell Signaling Technology). An anti-α-tubulin antibody (Proteintech, Chicago, IL, USA) was used as a loading control. After incubation with the horseradish peroxidase-conjugated secondary antibody (1:5,000; Proteintech), the chemiluminescence signal was detected using an enhanced chemiluminescence mixture (Sigma‑Aldrich), and images were captured using a gel imaging system (Bio‑Rad Laboratories, Inc., Hercules, CA, USA).

### Apoptosis, proliferation, migration, invasion and tube formation assays

Apoptosis was analysed using an Invitrogen Annexin V-FITC apoptosis detection kit following the manufacturer's instructions. Briefly, 1 × 10^6^ cells per well were seeded in 6-well plates. Next, the cells were harvested and stained with an FITC-conjugated anti-Annexin V antibody and propidium iodide after exosome treatment for 24 h. A flow cytometer (BD Biosciences, San Jose, CA, USA) was used to detect the stained cells. Cell proliferation was assessed using a CCK-8 assay (Dojindo Molecular Technologies, Inc., Rockville, MD, USA) according to the manufacturer's directions. Colony formation, cell migration and invasion assays were performed as previously described 12. For the tube formation assay, HUVECs were treated with exosomes for 48 h and then seeded in BD Matrigel for 6-8 h. Tube-like structures were imaged under a light microscope. For *in vitro* assays, 2 × 10^5^ recipient cells were incubated with 2 µg exosomes. GDC-0941 (200 nM) and MK-2206 (200 nM) were purchased from APExBIO.

**Mouse Studies** All animal experiments were performed in accordance with the National Institutes of Health (NIH) Guidelines for the Care and Use of Laboratory Animals and according to the protocols approved by the Animal Care and Use Committee at CSU. In the Matrigel plug assays, male BALB/c nude mice (6 to 8 weeks old) were randomly divided into four groups (A, B, C and D). For groups A and B, 9.5 µg of exosomes was mixed with 500 µL of Matrigel and EGF (150 ng/mL, Peprotech, SC, USA) and then subcutaneously injected. After 10 days, the Matrigel plugs were harvested. The level of angiogenesis is typically viewed by embedding and sectioning the plugs in paraffin and staining with Masson's trichrome, which stains the Matrigel blue and endothelial cells red 43. In each experiment, three random fields for each of the triplicate Matrigel plugs were quantitated using an image-capturing system linked to NIH Image. The results are expressed as the mean area occupied by the cells (μm^2^) ± SE. For groups C and D, HUVECs were pretreated with or without filipin III (1.5 μM), after which exosomes was added. Next, 300 µL of HUVECs (3 × 10^6^ cells) was mixed with 300 µL of Matrigel and then subcutaneously injected into the mice. On day 7 after the implantation, the Matrigel plugs were collected, fixed, sliced, and stained with eosin-hematoxylin. For the xenograft tumour model, a sample of 2 × 10^6^ HO-8910 cells was subcutaneously injected into the posterior flanks of BALB/c nude mice. After 9 days, 5 µg exosomes were injected into the xenografts in the posterior flank every other day for 1 week. Three days after the last injection, tumour tissues were dissected, fixed in 10% formalin, and embedded in paraffin for further study.

### Statistical analysis

Each experiment was repeated at least three times. Statistical analyses were carried out using SPSS version 18.0 (SPSS, Inc., Chicago, USA) and GraphPad Prism 5 (GraphPad Software, San Diego, CA, USA). Differences between the groups were analysed by Student's *t* test (unpaired, two-tailed) or one-way ANOVA. Pearson's correlation coefficient was applied to evaluate the relationship between miR-205 and MVD. Data are presented as the mean ± SD, *P < 0.05 **P < 0.01, and ***P < 0.001 were considered significant differences.

## Figures and Tables

**Scheme 1:**
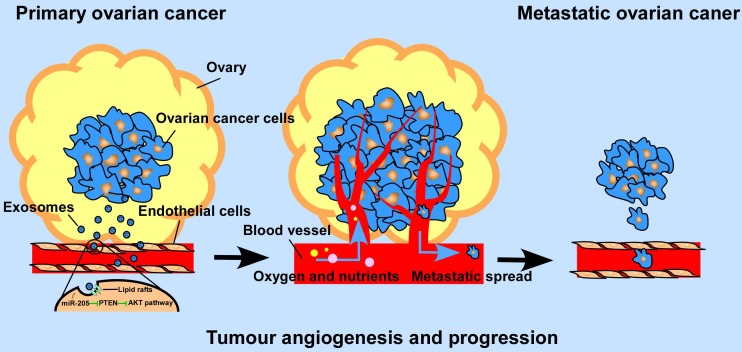
Schematic diagram of the role of OCC -secreted miR-205 in tumour angiogenesis.

## Abbreviations

CAV1: caveolin-1
CRC: colorectal cancer
DMEM: Dulbecco's modified Eagle medium
ECs: endothelial cells
EVs: extracellular vesicles
FBS: foetal bovine serum
HMG-CoA: 3-hydroxyl-3-methylglutaryl coenzyme A
HUVECs: human umbilical vein endothelial cell
ISH: *in situ* hybridization
IHC: immunohistochemistry
IOD: integral optical density
miRNAs: microRNAs
MVD: microvessel density
NPC: nasopharyngeal carcinoma
OC: ovarian cancer
OCCs: ovarian cancer cells
PBS: phosphate-buffered saline
TEM: transmission electron microscopy
SSC: saline sodium citrate
