# Supplementary material for: Ovarian cancer cell-secreted exosomal miR-205 promotes metastasis by inducing angiogenesis
Source: Theranostics. 2019 Oct 18;9(26):8206–20. doi: 10.7150/thno.37455 (PMC6857047; doi:10.7150/thno.37455)
Supplement: Supplementary file 1 — Supplementary figures and tables. [file thnov09p8206s1.pdf]

Table S1. Relative expression of mir-205 and MVD in OC tissues.

	Relative expression of miR-205	MVD
Median	0.229	34
Range	0.096-0.367	12-85
Mean±SD	0.228±0.063	40.625+19.539

Table S2. Clinical information of patients in the OC tissues.

Patient	Age, years	Sample receipt date, YMD	Stage	Metastasis (No:0; Yes:1)	Histological pattern
1	37	2015/3/24	3	1	Ovarian cancer
2	49	2017/3/15	1	0	Ovarian cancer
3	55	2017/4/11	3	1	Ovarian cancer
4	46	2017/5/23	2	1	Ovarian cancer
5	50	2017/5/23	3	1	Ovarian cancer
6	60	2017/6/9	1	0	Ovarian cancer
7	57	2017/6/13	4	1	Ovarian cancer
8	48	2017/6/20	3	1	Ovarian cancer
9	49	2017/6/20	2	1	Ovarian cancer
10	44	2017/9/29	1	0	Ovarian cancer
11	54	2017/10/31	3	1	Ovarian cancer
12	49	2017/11/2	4	1	Ovarian cancer
13	59	2017/11/7	4	1	Ovarian cancer
14	56	2017/11/16	1	0	Ovarian cancer
15	62	2017/11/30	1	0	Ovarian cancer
16	61	2017/12/1	1	0	Ovarian cancer
17	53	2017/12/12	2	1	Ovarian cancer
18	37	2017/12/12	2	1	Ovarian cancer
19	53	2017/12/12	3	1	Ovarian cancer
20	41	2017/12/19	2	1	Ovarian cancer
21	44	2017/12/19	2	1	Ovarian cancer
22	45	2017/12/22	3	1	Ovarian cancer
23	60	2017/12/26	3	1	Ovarian cancer

24	49	2018/1/3	1	0	Ovarian cancer
25	64	2018/1/26	2	1	Ovarian cancer
26	48	2018/2/2	2	1	Ovarian cancer
27	68	2018/2/9	2	1	Ovarian cancer
28	65	2018/2/9	2	1	Ovarian cancer
29	45	2018/2/23	1	0	Ovarian cancer
30	48	2018/3/6	2	1	Ovarian cancer
31	43	2018/3/8	2	1	Ovarian cancer
32	48	2018/3/14	3	1	Ovarian cancer
33	37	2018/3/15	2	1	Ovarian cancer
34	47	2018/4/8	3	1	Ovarian cancer
35	35	2018/4/8	3	1	Ovarian cancer
36	60	2018/4/24	3	1	Ovarian cancer
37	37	2018/4/28	3	1	Ovarian cancer
38	37	2018/4/28	3	1	Ovarian cancer
39	65	2018/5/3	3	1	Ovarian cancer
40	38	2018/5/11	3	1	Ovarian cancer
41	59	2015/6/11	-	-	Normal ovarian tissues
42	54	2016/1/12	-	-	Normal ovarian tissues
43	37	2016/5/26	-	-	Normal ovarian tissues
44	53	2016/10/27	-	-	Normal ovarian tissues
45	49	2017/3/15	-	-	Normal ovarian tissues
46	50	2017/3/15	-	-	Normal ovarian tissues
47	55	2017/4/11	-	-	Normal ovarian tissues
48	33	2017/9/20	-	-	Normal ovarian tissues
49	33	2017/9/20	-	-	Normal ovarian tissues
50	44	2017/9/29	-	-	Normal ovarian tissues
51	50	2017/12/19	-	-	Normal ovarian tissues
52	44	2017/12/19	-	-	Normal ovarian tissues
53	60	2017/12/26	-	-	Normal ovarian tissues
54	41	2018/4/3	-	-	Normal ovarian tissues
55	56	2018/4/27	-	-	Normal ovarian tissues
56	52	2018/4/27	-	-	Normal ovarian tissues
57	55	2018/4/28	-	-	Normal ovarian tissues
58	42	2018/5/8	-	-	Normal ovarian tissues
59	48	2018/5/18	-	-	Normal ovarian tissues
60	56	2018/5/23	-	-	Normal ovarian tissues
61	48	2018/5/29	-	-	Distant metastatic carcinoma

62	50	2014/12/26	-	-	Distant metastatic carcinoma
63	50	2017/11/17	-	-	Distant metastatic carcinoma
64	60	2018/04/24	-	-	Abdominal metastatic carcinoma
65	44	2018/04/28	-	-	Abdominal metastatic carcinoma
66	65	2018/05/04	-	-	Abdominal metastatic carcinoma
67	48	2018-02-02	-	-	Lymph node metastasic carcinoma
68	47	2017-11-10	-	-	Lymph node metastasic carcinoma

Table S3. Clinical information of OC patients and healthy volunteers in the circulating exosomes study.

Patient	Age, years	Sample receipt date, YMD	Metastasis (No:0; Yes:1)	Histological pattern
1	53	2017/12/19	0	Serum sample
2	58	2017/12/20	1	Serum sample
3	47	2017/12/26	0	Serum sample
4	38	2017/12/26	1	Serum sample
5	53	2017/12/27	1	Serum sample
6	47	2017/12/28	0	Serum sample
7	52	2018/1/18	0	Serum sample
8	49	2018/1/19	0	Serum sample
9	28	2018/02/02	healthy	Serum sample
10	29	2018/02/02	healthy	Serum sample
11	23	2018/02/02	healthy	Serum sample
12	25	2018/02/02	healthy	Serum sample
13	26	2018/02/02	healthy	Serum sample

Table S4. The sequences used in this study

Primer	Sequence (5'→3')

Cel-miR-239b-5p	UUUGUACUACACAAAAGUACUG
hsa-miR-205 mimic	UCCUUCAUUCCACCGGAGUCUG
hsa-miR-205 nc	UUUGUACUACACAAAAGUACUG
hsa-miR-205-5p inhibitor	CAGACUCCGGUGGAAUGAAGGA
PTEN-si1	GGAATATCTAGTACTTACT
PTEN-si2	GGTGTAATGATATGTGCAT
PTEN-si3	ACCGCCAAATTTAATTGCA
Flag-PTEN Forward	AGGCACTGGGCAGGTAAG
Flag-PTEN Reverse	CTGGAATAGCTCAGAGGC
PTEN Forward	CCTCAGCCGTTACCTGTGTG
PTEN Reverse	TCTGGATTTGACGGCTCCTCT

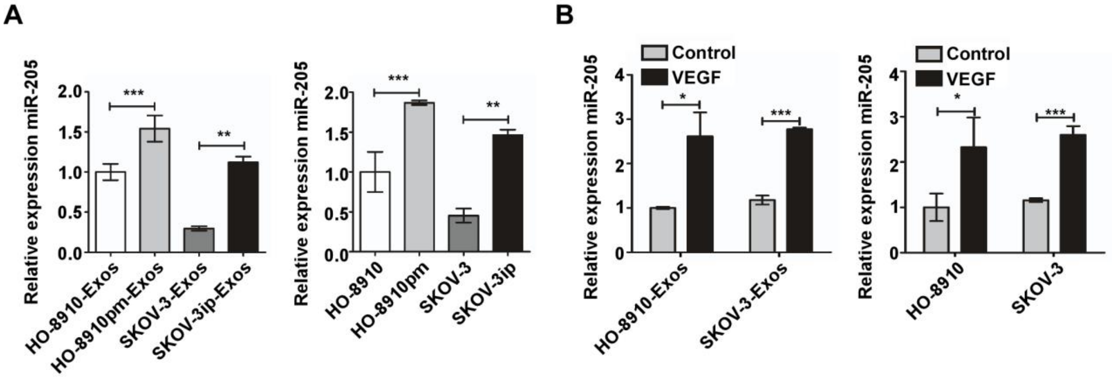

Supplymentary Figure S1: Expression level of miR-205 in cells can affect the expression levels of miR-205 in exosomes (**A**) The expression levels of miR-205 in OCCs and exosomes were measured by RT-PCR. (**B**) RT-PCR was used to detect the levels of miR-205 in cells and exosomes after treatment with $VEGF_{165}$. All data are shown as the mean ± SEM from at least three independent experiments. *$P < 0.05$, **$P < 0.01$ and ***$P < 0.001$, Student's *t* test.

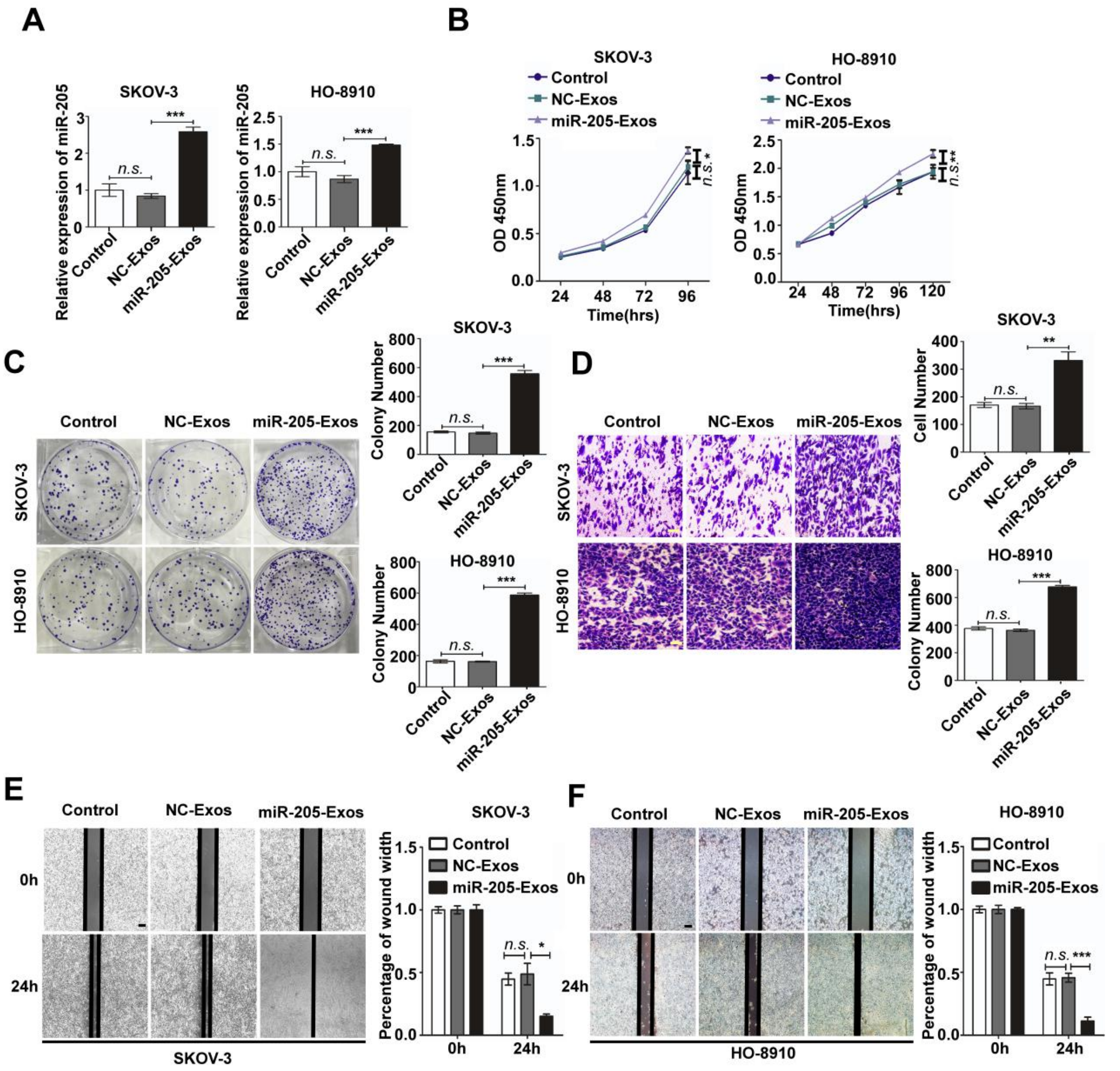

Supplymentary Figure S2: Exosomal miR-205 promotes invasion and metastasis in OCCs. (**A**) After treatment with the miR-205-Exos, NC-Exos and Control (blank control), the miR-205 levels of HO-8910 and SKOV-3 cells were measured by RT-PCR. (**B**) The cell proliferation of HO-8910 and SKOV-3 cells was measured by the CCK-8 assay after treatment with exosomes. (**C**) After incubated with exosomes, cell proliferation of HO-8910 and SKOV-3 cells was assessed by colony formation. (**D**) The transwell invasion assay was performed to measure the cell invasion ability of HO-8910 and SKOV-3 cells treated with exosomes. The scale bar represents 50 μm. (**E, F**) The wound closure assay was performed to measure the effect of exosomal miR-205 on the migration of OCCs. The scale bar represents 100 μm. All the in vitro experiments were repeated three times. The results are presented as the mean±SEM. Statistical significance was determined by a two-tailed, unpaired Student's t-test. *$P < 0.05$, **$P < 0.01$ and ***$P < 0.001$. *n.s*, not significant.

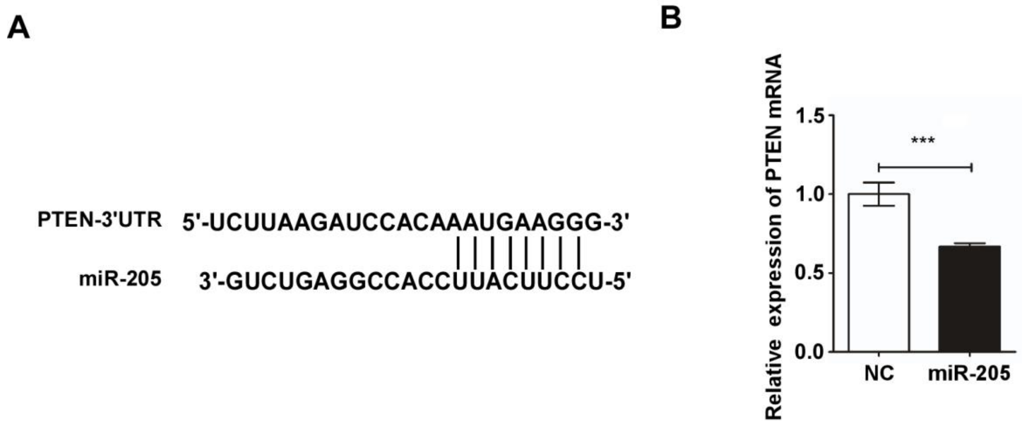

Supplymentary Figure S3: PTEN is the direct transcriptional target of miR-205. (**A**) Putative target sites for miR-205 in the 3′ UTR of PTEN. (**B**) Effects of miR-205 upregulation on endogenous PTEN mRNA level which were analyzed by qRT-PCR. The results are presented as the mean±SEM. Statistical significance was determined by a two-tailed, unpaired Student's t-test. ***$P < 0.001$.

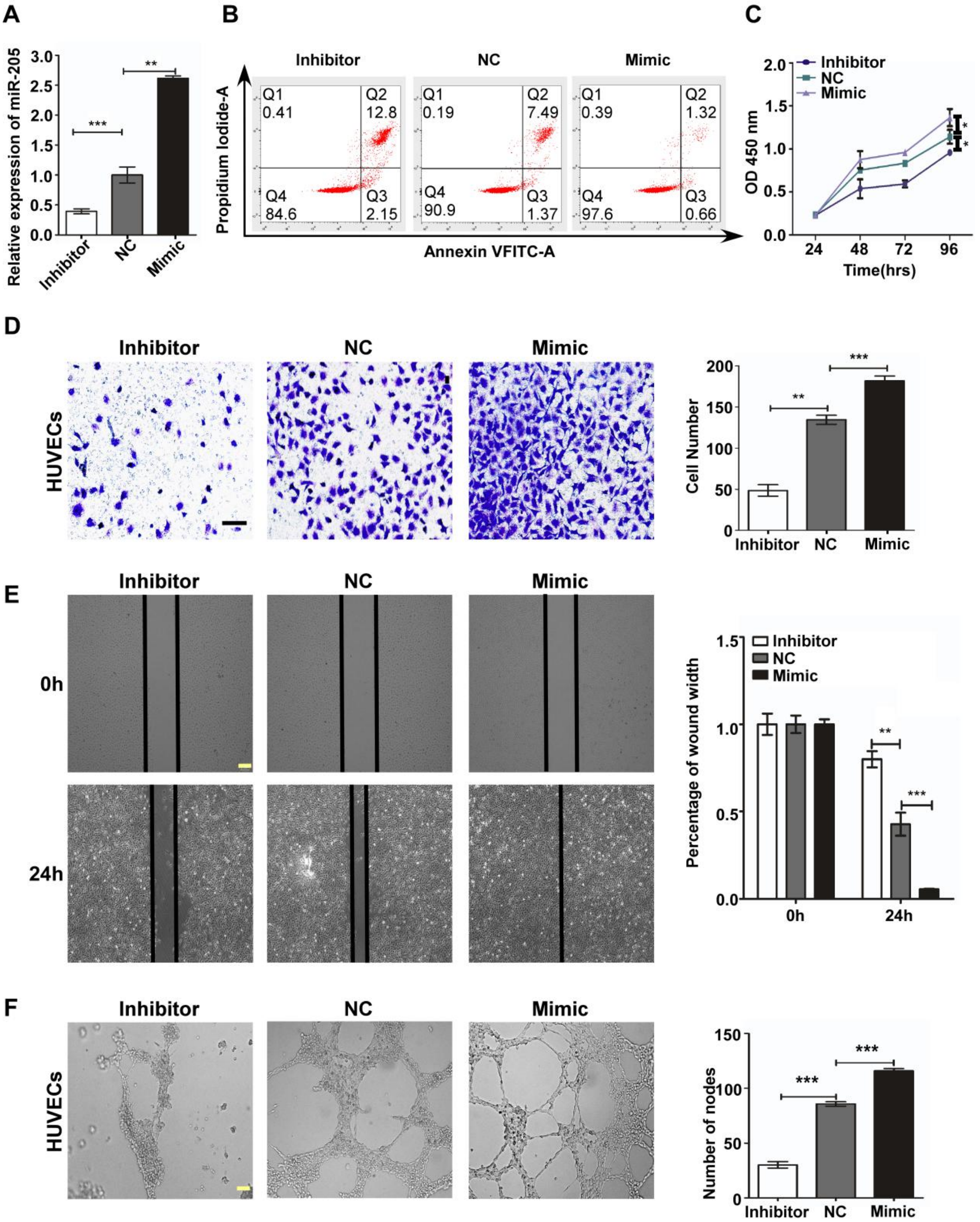

Supplymentary Figure S4. miR-205 regulates in vitro angiogenesis. (**A**) Transfection efficiency was measured by RT-PCR. (**B**) Analysis of apoptosis was performed at 24 h after transfection. (**C**) The CCK-8 assay was used to measure the effect of miR-205 on HUVEC cell growth. (**D**) Transwell assays were applied to measure cell migration. The scale bar represents 50 μm. (**E**) The wound closure assay showed cell migration by transfected HUVECs. The scale bar represents 100 μm. (**F**) The tube formation assay was performed with HUVECs after transfection. The scale bar represents 100 μm. All in vitro experiments were performed in triplicate and repeated three times. The results are presented as the mean ± SEM. Statistical significance was determined by a two-tailed,

unpaired Student's *t* test. *$P < 0.05$, **$P < 0.01$ and ***$P < 0.001$

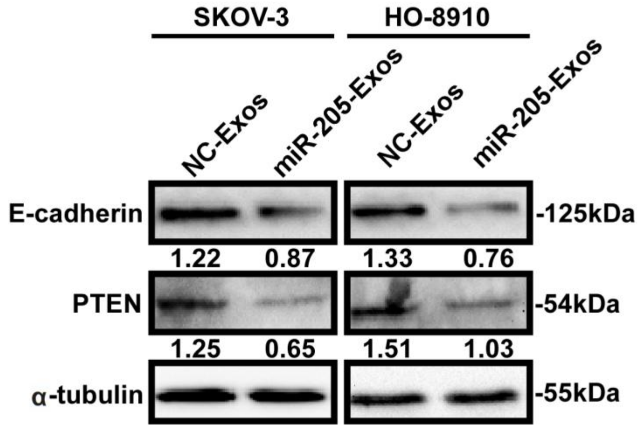

Supplymentary Figure S5: Exosomal miR-205 decreases the protein levels of PTEN and E-cadherin in OCCs.

(**A**) Western blotting analysis of E-cadherin, PTEN, α-tubulin in HO-8910 and SKOV-3 cells incubated with NC-Exos, miR-205-Exos.
